# Landscape analysis of alternative splicing in kidney renal clear cell carcinoma and their clinical significance

**DOI:** 10.18632/aging.205915

**Published:** 2024-06-10

**Authors:** Songtao Cheng, Zili Zhou, Jiannan Liu, Jun Li, Yu Wang, Jiantao Xiao, Yongwen Luo

**Affiliations:** 1 Department of Urology, Sichuan Provincial People’s Hospital, School of Medicine, University of Electronic Science and Technology of China, Chengdu, China; 2 Department of Gastrointestinal Surgery, Sichuan Provincial People’s Hospital, University of Electronic Science and Technology of China, Chengdu, China; 3 Department of Urology, Zhongnan Hospital of Wuhan University, Wuhan, China

**Keywords:** alternative splicing, kidney renal clear cell carcinoma (KIRC), splicing factor, prognosis, FMR1

## Abstract

A growing number of studies reveal that alternative splicing (AS) is associated with tumorigenesis, progression, and metastasis. Systematic analysis of alternative splicing signatures in renal cancer is lacking. In our study, we investigated the AS landscape of kidney renal clear cell carcinoma (KIRC) and identified AS predictive model to improve the prognostic prediction of KIRC. We obtained clinical data and gene expression profiles of KIRC patients from the TCGA database to evaluate AS events. The calculation results for seven types of AS events indicated that 46276 AS events from 10577 genes were identified. Next, we applied Cox regression analysis to identify 5864 prognostic-associated AS events. We used the Metascape database to verify the potential pathways of prognostic-associated AS. Moreover, we constructed KIRC prediction systems with prognostic-associated AS events by the LASSO Cox regression model. AUCs demonstrated that these prediction systems had excellent prognostic accuracy simultaneously. We identified 34 prognostic associated splicing factors (SFs) and constructed homologous regulatory networks. Furthermore, *in vitro* experiments were performed to validate the favorable effect of SFs FMR1 in KIRC. In conclusion, we overviewed AS events in KIRC and identified AS-based prognostic models to assist the survival prediction of KIRC patients. Our study may provide a novel predictive signature to improve the prognostic prediction of KIRC, which might facilitate KIRC patient counseling and individualized management.

## INTRODUCTION

As one of the most common malignancies, renal cell carcinoma (RCC) is accounting for 4.2% of newly diagnosed cancer cases. In 2019, there were about 73820 new cases with 14770 deaths from RCC in the United States [1, 2]. As the most common pathological type in RCC, kidney renal clear cell carcinoma (KIRC) represents 75–80% of all renal cancers and accounts for the majority of deaths from RCC [3]. Currently, there are about 30% of patients with distant metastasis when they are diagnosed [4]. Meanwhile, up to 30% of patients with localized tumor will continue to progress to metastasis [5]. These characteristics determine that KIRC has a poor prognosis. At present, robust and effective biomarkers to predict the prognosis of KIRC are still unavailable.

As a crucial post-transcriptional regulatory step, alternative splicing (AS) includes excision of introns and linking together of exons, which results in distinct mature mRNA transcript, and then translate into different proteins with different structures or functions [6]. It is reported that AS is responsible for 40% of protein modifications, and more than 95% of pre-mRNAs are alternatively spliced in mammals [7, 8]. Accumulating results show that AS is associated with carcinogenesis and tumor progression and provides effective prognostic value in various cancers [9–11]. For instance, as an important prognostic factor in early NSCLC, hMENA splice isoforms combined with clinical parameters could predict individual patient risk accurately [12]. P53δ is a novel p53 splice variant, which can predict the prognosis as a prognostic marker in ovarian cancer independently [13]. CDK12 modulates ALE splicing of ATM and a DNAJB6 isoform that promotes breast cancer cell invasion [14]. TGLI1, AS variant of GLI1, promotes glioblastoma angiogenesis and growth by targeting heparinase [15].

In our study, we tried to gain a deep insight into the AS landscape of KIRC and develop AS-associated prognostic signatures to improve the prognostic prediction of KIRC.

## RESULTS

### Landscape of AS events profiles in TCGA-KIRC cohort

**Figure 1:**
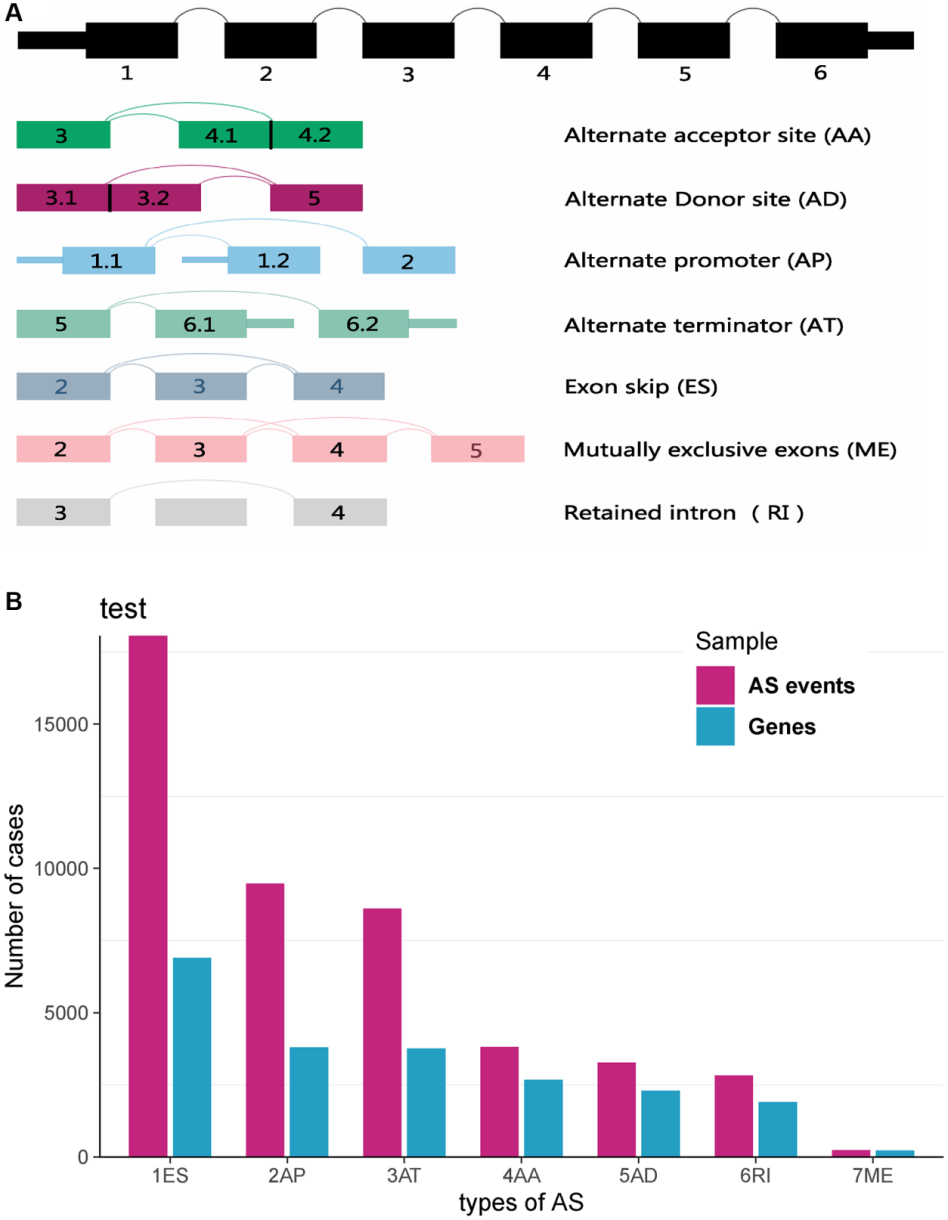
**Landscapes of AS events profiles in TCGA-KIRC dataset.** (**A**) Schematic diagram of seven types of AS events, that is AA (Alternate Acceptor site), AD (Alternate Donor site), AP (Alternate Promoter), AT (Alternate Terminator), ES (Exon Skip), ME (Mutually Exclusive Exons) and RI (Retained Intron). (**B**) Overview of AS events in TCGA-KIRC dataset.

Comprehensive AS events profilings of 533 KIRC patients were obtained from TCGA-KIRC cohort. All AS events were divided into seven types, which are ES, AP, AT, AA, AD, RI, and ME. Separately, the seven types of special splicing patterns were shown in Figure 1A. According to integrated analysis of SpliceSeq tool, we identified 46276 AS events from 10577 genes, comprising of 18062 ESs in 6899 genes, 9472 APs in 3793 genes, 8611 ATs in 3762 genes, 3810 AAs in 2676 genes, 3265 ADs in 2295 genes, 2821 RIs in 1897 genes, and 235 MEs in 227 genes (Figure 1B). As a result, we have known that each gene may account for more than one AS event. And the results showed that ES ranks first in all AS events, then comes the AP and AT.

### Prognostic-associated AS events in TCGA-KIRC cohort

**Figure 2:**
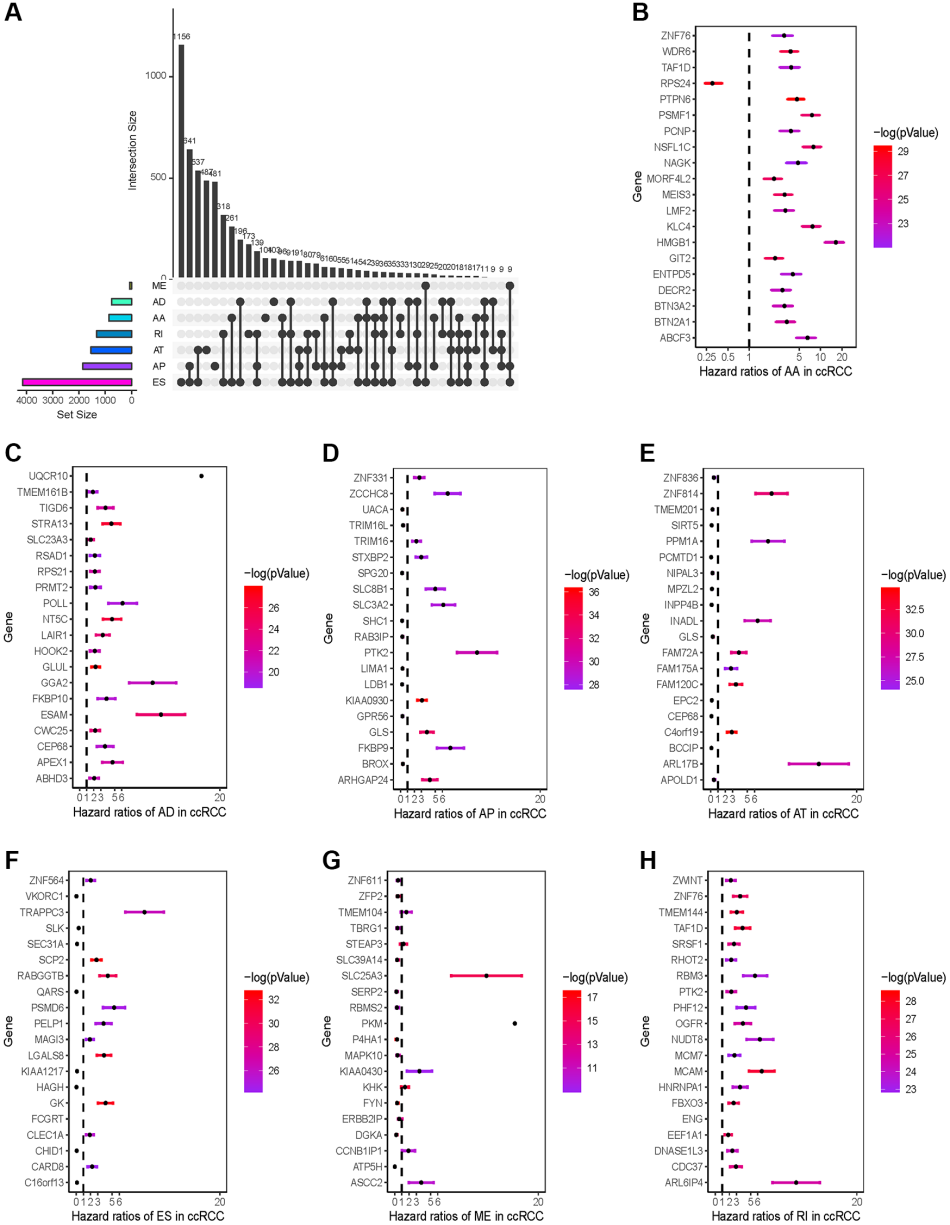
**Prognosis-related AS events profiles.** (**A**) Intersection UpSet plot of seven types of prognosis-related AS events in KIRC. (**B**–**H**) Forest plots of HRs of the top 20 prognostic-associated seven types of AS events. The color scale on the right side indicates the *P*-value.

AS events were related to carcinogenesis, progression and prognosis in a variety of tumors. Therefore, we conducted Cox regression analysis to identify prognostic-associated AS events of KIRC. As a result, 3823 ES events in 2504 genes, 3587 AP events in 1853 genes, 3048 AT events in 1546 genes, 1002 AA events in 866 genes, 887 AD events in 755 genes, 1186 RI events in 868 genes, and 75 ME events in 72 genes were associated with KIRC prognosis. ES was the most frequent AS event related to OS, followed by AP and AT. Among them, more than half were unfavorable predictors, and the others were favorable. The result was shown as an upset plot (Figure 2A). According to the results, we found that one single gene had more than one AS type. The ES was also the most common prognostic AS type. The top 20 prognostic AS events related genes of seven AS patterns were visualized in forest plots (Figure 2B–2H).

### Pathway and functional enrichment analysis

**Figure 3:**
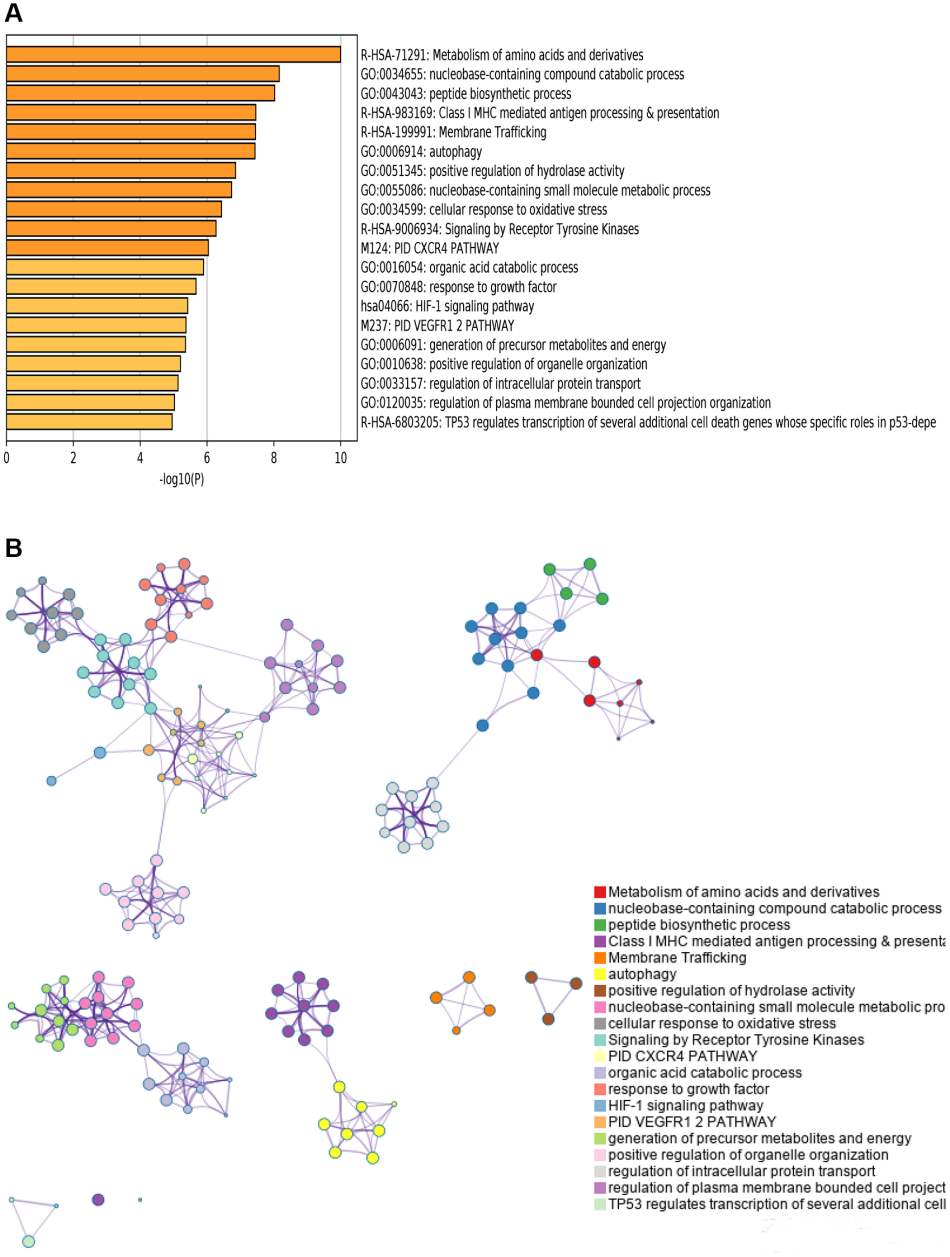
**Pathway and functional enrichment analysis.** (**A**) Top 20 pathways and functional enrichment clusters. (**B**) The 20 MCODE of genes from prognostic-related AS events.

We utilized Metascape to identify the enriched pathways of prognostic associated AS to conduct a deep investigation of the underlying mechanism of prognostic associated AS genes in KIRC. The pathway and functional enrichment analysis indicated that metabolism related pathways were the most frequently involved, including “Metabolism of amino acids and derivatives”, “nucleobase-containing compound catabolic process”, “peptide biosynthetic process”, etc. (Figure 3A). Protein-protein interaction network analysis revealed that these AS genes were concentrated in twenty MCODE components (Figure 3B).

### Development of AS-based predictive model

**Figure 4:**
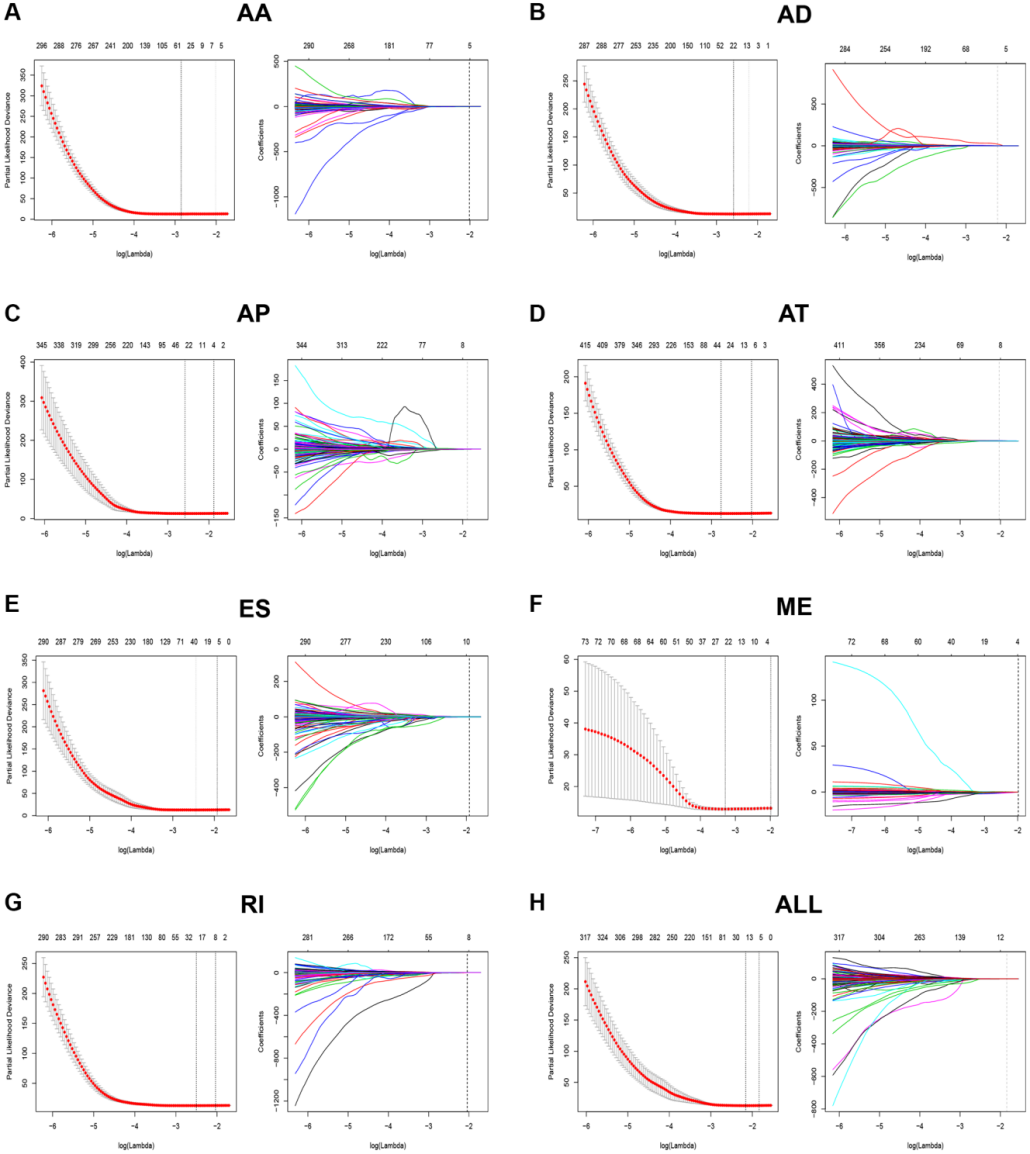
**LASSO Cox model to construct prognostic-related AS signatures.** (**A**–**H**) Indicated constructions of the most valuable prognostic-related AS signatures and the LASSO coefficients profiles of seven types of AS events. The vertical lines were drawn at the optimal values by the minimum criteria and the 1-SE criteria.

**Figure 5:**
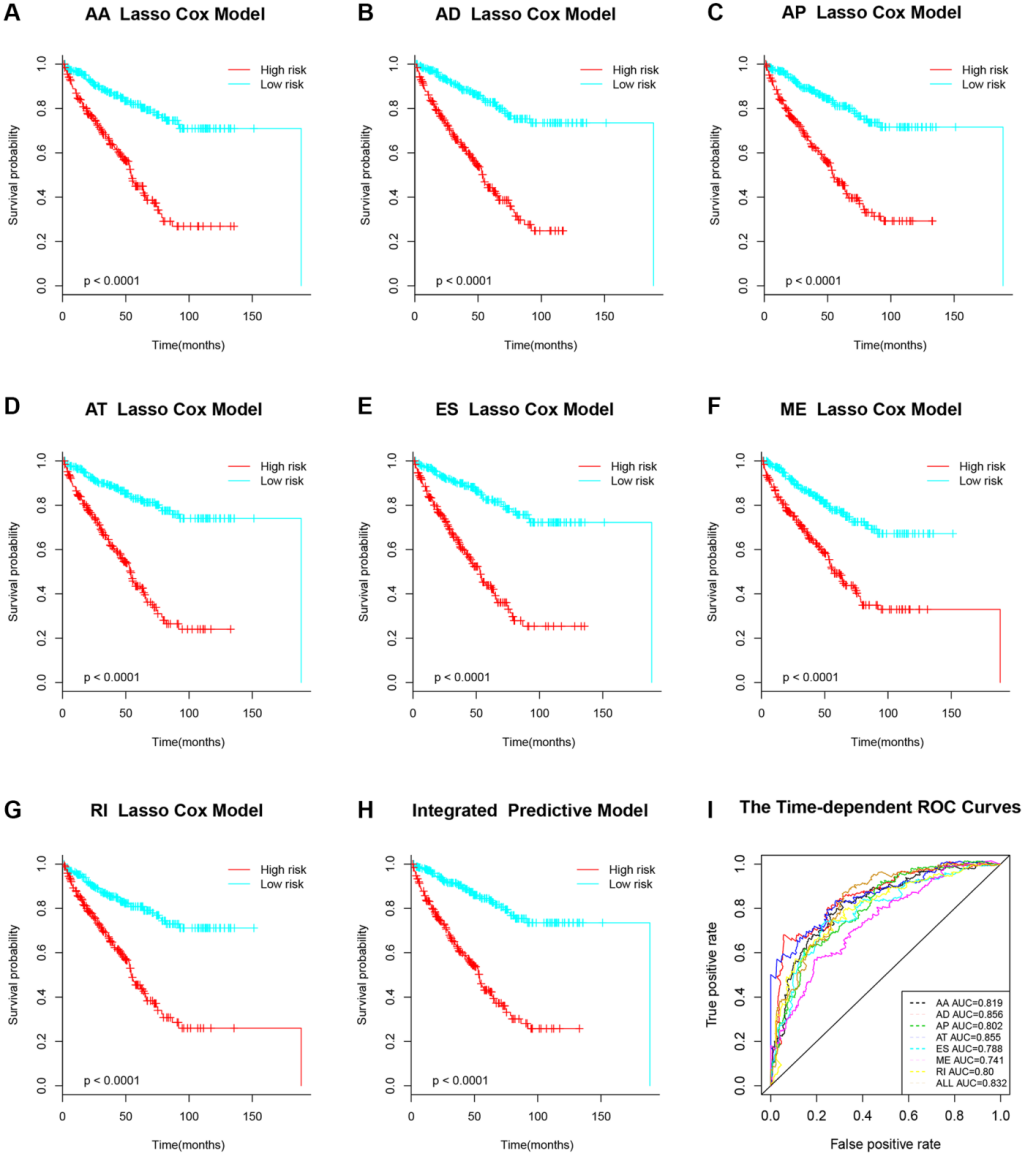
**Kaplan-Meier survival analysis of prognostic-related AS signature.** (**A**–**H**) Survival analysis of seven types of prognostic-related AS signature. (**I**) Time-dependent ROC for different survival prediction models.

Because of the high mortality of KIRC, we aimed to develop an AS-associated prediction model to improve prognostic prediction of KIRC. To construct the prediction model precisely, we performed LASSO Cox regression analysis to identify the most optimal prognostic AS model of seven types of AS patterns. As shown in Figure 4, we figured out the optical lambda (Figure 4A–4H) from the seven types of AS and established clinical prediction models. Then, according to the median risk score of prediction models, patients were divided into either low or high-risk groups. And those in the high-risk group had poorer survival compared with the ones in the low-risk group from the Kaplan–Meier survival analysis (Figure 5A–5H). By using time-dependent ROC analysis, we evaluated the predictive accuracy of AS signatures. The time-dependent ROC analysis results showed that integrated AS signatures, AD predictive signatures, and AT predictive signatures had better performance than other AS signatures (Figure 5I). All these results showed that these AS signatures could be a reliable and robust predictor of prognosis for KIRC patients.

### Network of prognostic-associated alternative splicing events

**Figure 6:**
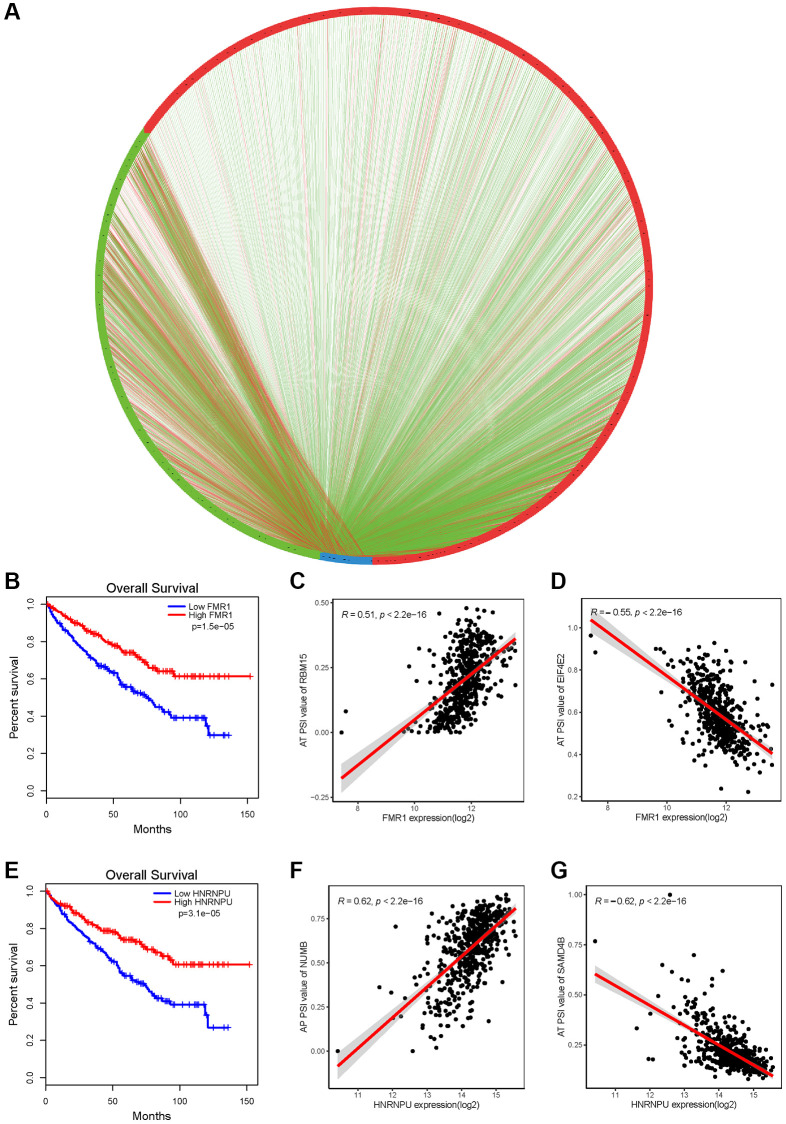
**AS correlation network in KIRC.** (**A**) Splicing correlation network in KIRC. The expression of all the prognostic-related splicing factors (blue dots) was negatively (green line) or positively (red line) associated with PSI value of all the favorable prognosis and adverse AS events (red dots and green dots, respectively). (**B**, **E**) Using the GEPIA tool to analyze the prognosis of splicing factors FMR1 and HNRNPU, respectively. (**C**, **D**, **F**, **G**) Representative dot plots of correlations between expression of SFs FMR1 or HNRNPU and PSI value of AS events (*P* < 0.001).

Given all AS alteration patterns were broadly regulated by critical splicing factors (SFs), thus, we identified the prognostic-related SFs and explored the correlations between prognostic-related SFs and AS events. Univariate Cox regression analysis showed that 30 SFs were related to the survival of KIRC patients significantly (Supplementary Table 1). Most of the SFs predicted good outcomes of patients, such as FMR1 and HNRNPU (Figure 6B, 6E). Furthermore, analysis of correlation revealed that a total of 1369 prognostic-associated AS events were correlated to 23 prognostic-associated SFs significantly in KIRC patients (Supplementary Table 2). The results of correlation analyses were visualized with a splicing regulatory network (Figure 6A). Interestingly, we found that most of the prognostic-related SFs were positively (red lines) related to the adverse prognostic AS events (green dots) and negatively (green lines) related to favorable prognostic AS events (red dots), Representative correlations between SFs and specific AS events were presented in dot plots. For example, expression of FMR1 was positively correlated with AT of RBM15 (Figure 6C), and negatively correlated with AT of EIF4E2 (Figure 6D), while expression of HNRNPU was positively correlated with AP of NUMB (Figure 6F), and negatively correlated with AT of SAMD4B (Figure 6G).

### Favorable effect of FMR1 on KIRC

**Figure 7:**
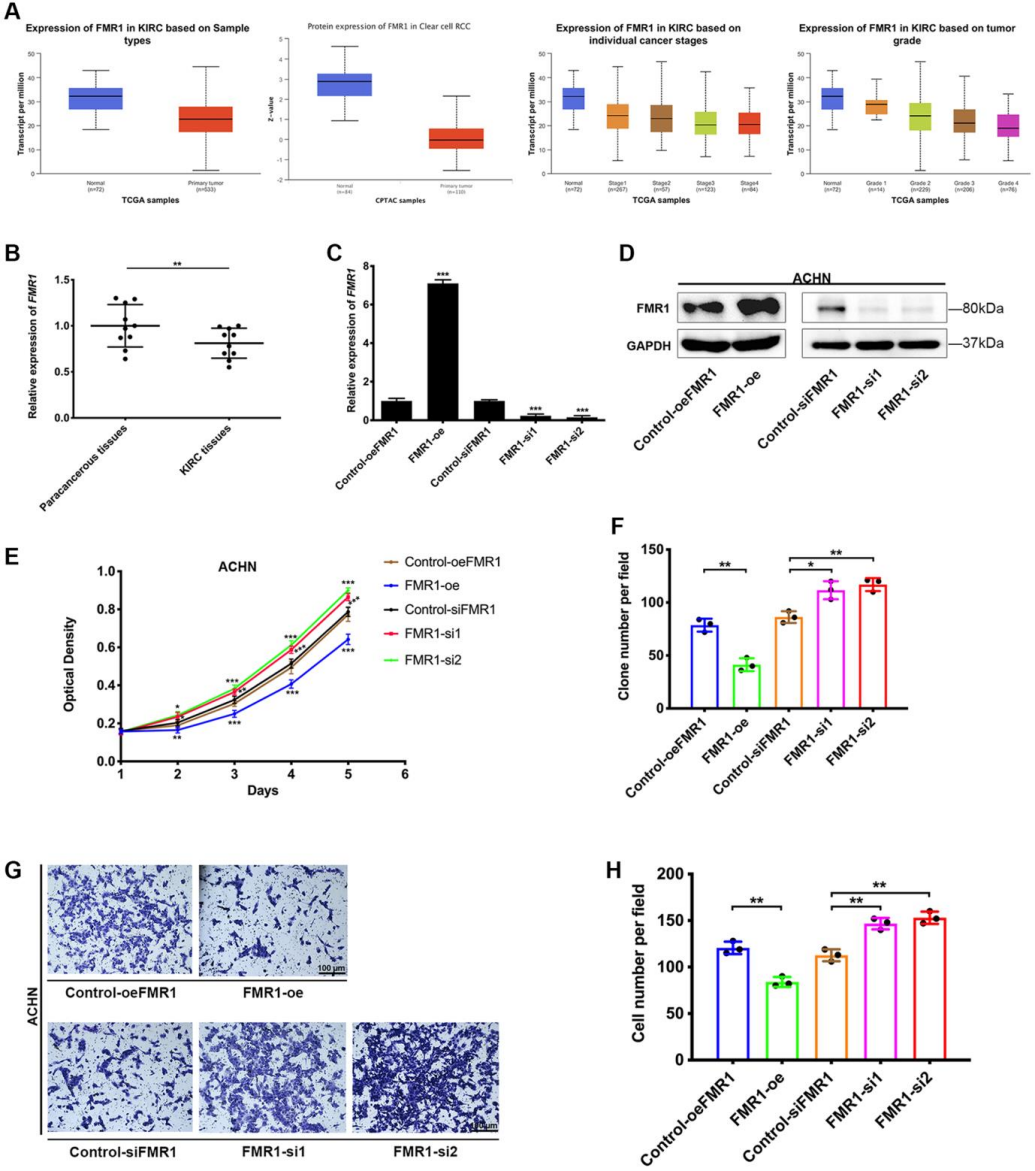
**Protective effect of FMR1 in KIRC.** (**A**) The expressing level of FMR1 in KIRC and normal tissues. (**B**) The qRT-PCR detects FMR1 expression of paired paracancerous tissues and KIRC cancer tissues from Zhongnan Hospital. (**C**) Overexpression and knockdown efficiency in ACHN cells. (**D**) WB confirmed the overexpression and knockdown of FMR1 in ACHN cells. (**E**) MTT assay to investigate the proliferation of ACHN cells. (**F**) Clonogenic formation results from three independent experiments. (**G**) Transwell migration assay to investigate cell migration ability, scale bar = 100 μm. (**H**) Confirmed by statistical analysis. ^*^p < 0.05, ^**^p < 0.01, ^***^p < 0.001.

To further validate the good outcomes of SFs in KIRC, we conducted in vitro experiments with KIRC tissues and cell lines. The results from TCGA and CPTAC databases indicated that FMR1 is downregulated in KIRC tissues compared to normal tissues both at protein and gene levels, and the expression of FMR1 is negatively related to stage and grade of KIRC (Figure 7A). Consistently, our clinical samples also verified that FMR1 expression is reduced in KIRC tissues compared to paracancerous tissues (Figure 7B). So, we chose FMR1 for the next experiments. FMR1 was overexpressed by transfecting FMR1 plasmid, and was knockdown by transfecting FMR1-target-siRNAs separately in ACHN cell. The overexpression and knockdown efficiency were confirmed by qRT-PCR (Figure 7C) and WB (Figure 7D). After overexpression of FMR1, the cell proliferation was significantly suppressed (Figure 7E). Furthermore, clonogenic survival ability of ACHN was also inhibited by FMR1 (Figure 7F). On the contrary, FMR1 knockdown promoted ACHN cells proliferation and clonogenic formation efficiency (Figure 7E, 7F). Cell migration contributed to tumor progression. Then, we conducted transwell migration assay, which showed that FMR1 overexpression could reduce ACHN cell migration rate, while FMR1 knockdown promoted the migration, compared to corresponding control group (Figure 7G). Statistical analysis of 3 independent experiments confirmed the results (Figure 7H). Afterall, our results revealed the favorable effect of SFs FMR1 on KIRC, but the underlying genetic mechanisms still need to be further explored.

## DISCUSSION

AS variants play key roles in tumor progression and oncogenesis [12–15]. In the current study, we deeply explored alternative mRNA splicing events based on TCGA-KIRC database. In total, we detected 46276 AS events from 10577 genes. Among them, the most common AS type was ES, then comes the AP, AT, AA, AD, RI and ME. Owing to the high mortality of KIRC, we determined to figure out prognosis-related AS events. Through pathway enrichment analysis, we found that metabolism pathways were the most enriched pathways in these genes of prognosis-related AS events. Furthermore, AS-based prognostic signatures were carried out, and we found that AS events have great value in assessing the prognosis in KIRC patients. AS is a critical component of the regulation of gene expression pathways in multicellular organisms, and it is strictly regulated by SFs. Previous researches based on the TCGA database have revealed that mutations in gene-encoding SFs were extensively related to specific cancer types [16–18]. We developed a network of prognostic-associated alternative splicing events. As a result, we can see that AS-SFs networks indeed exert a key effect in the regulation of the occurrence of KIRC.

AS patterns are dramatically different and contribute significantly to the identity, development, and diversity of cells, tissue, and organs. Besides, AS events are involved in many life processes, including cellular proliferation, differentiation, necrosis and tissue formation [18, 19]. In cancer, AS could promote the progression of primary tumor cells in many aspects, including greater proliferation capacity, stronger invasive property, and appearance of drug resistance, which could lead to tumor heterogeneity that is a tricky tissue for cancer treatment [20–23]. Meanwhile, growing evidence revealed that AS plays a vital role in the progression and initiation of KIRC. For instance, pVHL172 is translated from variant 2 by AS of exon 2. Instead of acting as a tumor suppressor compared to other isoforms, pVHL172 induces the aggressiveness of renal tumors [24]. PTBP1 promotes KIRC proliferation, migration and invasion through regulating AS of PKM [25]. EZH2 exon 14, which is alternatively spliced by SF3B3, inhibits cell growth, proliferation, migration, and tumorigenicity in a KIRC xenograft model, while EZH2 has the opposite effect [26]. There are many other cases showing the oncogenic effects of AS events in KIRC. In the current research, we also found that more than half of AS events were unfavorable predictors in KIRC. In all, we explored the correlations of AS and SFs and developed AS-based prognostic signatures in KIRC, which provides the implications of potential cancer biomarkers and potential therapeutic targets.

To significantly reduce KIRC mortality, more prognostic biomarkers are urgently needed. The results from some previous researches indicated some novel prognostic kinds of signatures for KIRC, including exosomes and some kinds of non-coding RNAs [27–30]. Benefiting from great achievements of next-generation sequencing techniques, the access to genome and cDNA sequences, microarrays, and high-throughput cDNA sequencing leads to the genome-wide assessment of transcripts, which can provide further insights into expressions and patterns of the genome [31, 32]. TCGA database provides us with a diversity of resources for cancer biomarkers search at the genomic level. We also chose one of the SFs FMR1, which is positively related to the survival rate of KIRC patients, negatively related to the proliferation and migration of KIRC cell ACHN, and downregulated in KIRC tissues. Our results indicated that FMR1 or other SFs could be potential biomarkers in future researches. But the mechanisms of how FMR1 or other SFs regulate or interact with AS genes and event still need to be further explored. Our splicing regulation network between AS events and SFs may give us a new insight into underlying genetic mechanisms of oncogenesis and progression of KIRC.

However, this study has some limitations. Firstly, all the data of this study were obtained from publicly available database. Some important clinical information was not available to us, which might serve to bias our results. Second, this is a retrospective study, a multicenter and prospective study is needed to validate our results. Finally, further research is needed to elucidate molecular mechanisms of AS regulation.

In conclusion, we proved that prognostic-associated AS events could be applied to predict the survival risks in KIRC patients. It may possess great potential value in clinical practice. AS-SFs system is complex, and deep researches should be carried out to comprehensively analyze the interaction networks of AS.

## CONCLUSIONS

In conclusion, we overviewed AS events in KIRC and identified AS-based prognostic models to assist the survival prediction of KIRC patients. Our study may provide a novel predictive signature to improve the prognostic prediction of KIRC, which might facilitate KIRC patients counseling and individualized management.

## METHODS

### Data acquisition and curation process

RNA-seq raw counts of kidney renal clear cell carcinoma (KIRC) and corresponding clinical data were achieved from The Cancer Genome Atlas (TCGA) (https://tcga-data.nci.nih.gov/tcga/). Then, SpliceSeq tool, a Java application, was used to analyze the mRNA splicing patterns of KIRC patients. Rating from zero to one, the Percent Spliced In (PSI) value was employed to evaluate AS events and calculate for seven types of alternative splicing events: Exon Skip (ES), Alternate Promoter (AP), Alternate Terminator (AT), Alternate Acceptor site (AA), Alternate Donor site (AD), Retained Intron (RI) and Mutually Exclusive Exons (ME). AS events with PSI value >75% were included for all analysis.

### Prognostic associated AS events identification

To identify all prognostic AS events in KIRC, 537 KIRC patients in total with overall survival (OS) data were collected. The corresponding clinical information was extracted and summarized. Then, the univariate Cox regression was taken to identify prognostic factors of seven types of AS patterns. We used a novel visualization tool UpSet plot [33] to quantitatively analyze the interactive sets, to visualize intersections of seven types of prognostic associated AS events.

### Gene functional enrichment analysis

We conducted pathway enrichment analysis of genes of survival-associated AS to detect the underlying mechanisms of survival associated alternative splicing genes in KIRC. Metascape (http://metascape.org/) is a web portal for gene analysis and annotation. We used the tools in this portal to search for deep insight into the biological functions of survival-associated AS genes [34]. The automated meta-analysis tool provided in this portal could be used to detect unique and common pathways within a group of orthogonal target-discovery studies. And the protein-protein interaction (PPI) analysis was also accessible based on BioGrid, enrichment heatmaps generation and interactive visualization of Gene Ontology (GO) networks.

### Establishment of predictive models by LASSO Cox regression

By conducting Cox regression model with LASSO (least absolute shrinkage and selection operator) penalty, the LASSO Cox regression analysis could simultaneously achieve shrinkage and variable selection. We used LASSO Cox regression model to establish the most predictive models of KIRC based on AS events. To prove if the predictive models can make a difference between the long OS patients and the shorter OS ones, Kaplan–Meier curves were performed. And the receiver operator characteristic curves were performed to further evaluate the efficiencies of each predictive model by running survivalROC package in R.

### Construction of splicing factor regulatory network

All the human splicing factors (SFs) were achieved from the SpliceAid database (http://www.introni.it/splicing.html) [35]. EdgeR package in R software was performed for normalized expression profiles of SFs in TCGA-KIRC dataset [36]. The expression values of SFs were log2(* + 1)-transformed for further analysis. We then conducted univariate Cox regression analysis to determine SFs associated with survival. Next, Pearson correlation analysis was conducted between the PSI value of AS events, which were obtained from previous prognostic signature construction, and the expression level of prognosis-associated SFs. Then, with a criterion of adjusted *p* < 0.05, we selected the significant correlation pairs and used Cytoscape (version 3.5.1) to construct the potential SF-AS regulatory network.

### Ethical statement of KIRC tissues

Ten paired KIRC tumor and paracancerous tissues were obtained through surgery in Zhongnan Hospital of Wuhan University, and patients’ clinicopathological characteristics are detailed in Supplementary Table 3. The fresh tumors and paracancerous tissues were immediately immersed in liquid nitrogen for subsequent experiments. The pathological diagnosis of KIRC tumor tissues specimens were independently validated by two pathologists. All patients had signed informed consents before the study. Using of human KIRC tissues was approved by the Ethics Committee of Zhongnan Hospital of Wuhan University (Approval No. 2020102).

### Cell culture and transfections

Human KIRC cell lines ACHN was provided by Chinese Academy of Sciences, China. MEM supplemented with 10% fetal bovine serum was used to cultivate the cells. We purchased siRNA and plasmid from GenePharma (China). The sense sequences were as follows: FMR1-si1, 5′-GTGTTAGTGGCTTCATCAGTT-3′; FMR1-si2, 5′-GCCTGATAGGCAGATTCCATT-3′; Control-siFMR1, 5′-UUCUCCGAACGUGUCACGUTT-3′. Cell transfection was mediated by Lipofectamine 2000 with either plasmid or siRNA.

### RNA extraction and qRT-PCR

Human KIRC tumor and paracancerous tissues, and ACHN cells were taken to extract cell total RNA by following the instruction manual of Qiagen RNeasy Kit (Cat. #74101). The RNA was quality controlled and reverse transcribed to cDNA. qRT-PCR was then conducted using iQ™ SYBR^®^-Green Supermix (Bio-Rad, USA). The primer sequences were as follows: FMR1 forward primer, 5′-CCAACAAACCTGCCACAAAAG-3′, reverse primer, 5′-GCACACATTTGCCGTAAGTCTT-3′; GAPDH forward primer, 5′-TGCACCACCAACTGCTTAG-3′, reverse primer, 5′-GATGCAGGGATGATGTTC-3′.

### Protein extraction and WB

ACHN cells were lysed in RIPA buffer for 30 minutes on ice. After spinning for 15 minutes at 4°C, the total protein included in the supernatant was collected and boiled in water bath for denaturation. Then, the protein band was separated and detected as we previously described [37].

### Cell phenotype experiment

After transfected with plasmid or siRNA, 3000/well cells were seeded into 96-well plates. Cell absorbance at 490 nm was measured every single day after treated with MTT and DMSO to evaluate cell viability. Similarly, 1000/well cells were seeded into 6-well plates for 10–14 days to perform clonogenic formation assay. The clone number was counted after the colony get fixed and stained with crystal violet.

For transwell migration assay, 3 × 10^4^ cells were seeded into the upper chamber with serum-free medium. Cells migrated to the lower chamber containing serum medium from the upper chamber after 24 hours incubating. Then, the cells were fixed, stained and counted for statistical analysis.

### Statistical analysis

R software 3.5.0 was used to conduct all the statistical analyses. Two-tailed Student’s *t*-test was chosen to investigate if there is a statistical difference between two groups. The cutoff probability value was set at *P* < 0.05. χ2 test was used to analyze correlations between AS events and clinicopathological parameters. As to the survival differences, we performed Kaplan-Meier survival analysis between the low-/high-risk groups. By running survival package in R, we conducted a two-sided log-rank test. And by running survivalROC package in R, the time-dependent receiver operating characteristic (ROC) analysis was conducted to detect the prediction accuracy of the predictive model.

### Availability of data and materials

Raw data were deposited in The Cancer Genome Atlas (TCGA) (https://tcga-data.nci.nih.gov/tcga/). The data are available from the corresponding author upon reasonable request.

## Supplementary Materials

Supplementary Tables 1 and 3

Supplementary Table 2
