# Supplementary material for: Landscape analysis of alternative splicing in kidney renal clear cell carcinoma and their clinical significance
Source: Aging (Albany NY). 2024 Jun 10;16(11):10016–32. doi: 10.18632/aging.205915 (PMC11210227; doi:10.18632/aging.205915)
Supplement: Supplementary Tables 1 and 3 [file aging-16-205915-s001.pdf]

SUPPLEMENTARY TABLES

Supplementary Table 1. Univariate Cox regression analysis of 30 prognosis-associated SFs in KIRC.

Gene	HR	Z	*p*-value	Lower	Upper
FMR1	0.621467917	−2.732589657	0.006283857	0.441821883	0.874158538
SRSF11	1.685010272	3.632577011	0.000280605	1.271565409	2.232885227
HNRNPL	4.007894656	3.844879191	0.000120612	1.975031114	8.133147602
HNRNPU	0.514504213	−3.357625669	0.00078615	0.349073214	0.758335429
HNRNPM	0.556213478	−3.307673673	0.000940744	0.392901976	0.78740615
HNRNPF	0.609952908	−2.425104282	0.01530399	0.409047036	0.909534888
RBFOX2	0.692545956	−2.488846599	0.012815825	0.518563616	0.924900796
SF1	0.634492442	−2.465594068	0.013678625	0.441946981	0.910925238
DAZAP1	2.954170151	5.746926637	9.09E-09	2.04171703	4.274402943
HNRNPH2	0.567871474	−2.84005707	0.004510546	0.384285946	0.839161604
HNRNPA0	0.477406745	−3.581171098	0.000342058	0.318526256	0.715536619
NOVA2	0.772336454	−3.713899717	0.00020409	0.673903698	0.885146646
TRA2A	2.282696947	5.001379783	5.69E-07	1.651877685	3.154413548
RBM5	1.497541895	3.025403637	0.002483015	1.152821971	1.945340898
RBM4	0.581450677	−2.257988013	0.023946407	0.363167429	0.930933952
RBMX	0.473821151	−3.514962026	0.000439817	0.312417321	0.71861087
KHDRBS1	0.436892796	−4.195827154	2.72E-05	0.296746777	0.643226246
KHDRBS3	0.673100149	−4.188333841	2.81E-05	0.559278616	0.810086059
KHDRBS2	1.12877534	2.156502266	0.031044467	1.01110095	1.260144963
SRSF4	2.198858748	3.49531939	0.000473495	1.413558342	3.420431722
SRSF7	2.195549812	3.600427916	0.000317694	1.430926947	3.368752673
SRSF6	1.406302941	2.136616065	0.032629232	1.0285915	1.922714667
SRSF1	2.321532401	2.906359483	0.003656611	1.315548722	4.096779236
SRSF3	0.570910124	−2.194755494	0.028181142	0.346080788	0.941798508
ZRANB2	1.741171224	3.581635277	0.00034145	1.285425047	2.358501757
ELAVL2	0.841793727	−3.315809252	0.000913782	0.760317542	0.93200096
HNRNPA2B1	3.20577654	4.615325615	3.92E-06	1.954706074	5.257569596
PCBP1	0.741119454	−3.985142818	6.74E-05	0.639583023	0.858775211
HNRNPLL	0.581399495	−3.05077438	0.00228252	0.410357007	0.823734862
TIA1	1.809131386	4.769843249	1.84E-06	1.417991835	2.308163059

Supplementary Table 3. Clinicopathological characteristics of ten KIRC samples.

Sample	Age	Gender	Grade	Stage
1	54	Female	3	I
2	56	Male	3	III
3	62	Male	3	II
4	59	Male	3	I
5	72	Male	2	I
6	65	Female	2	I
7	57	Male	2	II
8	46	Male	2	II
9	84	Female	2	I
10	56	Male	2	I
